# Low-Calorie, High-Protein Ketogenic Diet Versus Low-Calorie, Low-Sodium, and High-Potassium Mediterranean Diet in Overweight Patients and Patients with Obesity with High-Normal Blood Pressure or Grade I Hypertension: The Keto–Salt Pilot Study

**DOI:** 10.3390/nu17101739

**Published:** 2025-05-20

**Authors:** Matteo Landolfo, Lucia Stella, Alessandro Gezzi, Francesco Spannella, Paolo Turri, Lucia Sabbatini, Sofia Cecchi, Beatrice Lucchetti, Massimiliano Petrelli, Riccardo Sarzani

**Affiliations:** 1 Clinical and Molecular Sciences Department, Centre of Obesity, “Politecnica delle Marche” University, 60127 Ancona, Italy; m.landolfo@univpm.it (M.L.); a.gezzi@inrca.it (A.G.); p.turri@inrca.it (P.T.); l.sabbatini@inrca.it (L.S.); r.sarzani@univpm.it (R.S.); 2 Clinical Medicine and Geriatrics, “Hypertension Excellence Centre” of the European Society of Hypertension, Istituto di Ricovero e Cura a Carattere Scientifico (IRCCS)—Istituto Nazionale di Ricerca e Cura Anziani (INRCA), 60127 Ancona, Italy; 3 Endocrinology and Metabolic Disease Clinic, Azienda Ospedaliero-Universitaria delle Marche, 60127 Ancona, Italy; l.stella@pm.univpm.it (L.S.); soficecchi@gmail.com (S.C.); b.lucchetti@pm.univpm.it (B.L.); massimiliano.petrelli@ospedaliriuniti.marche.it (M.P.)

**Keywords:** ketogenic diet, Mediterranean diet, high blood pressure, hypertension, bioelectrical impedance analysis, ambulatory blood pressure monitoring

## Abstract

**Background and Objective:** Dietary interventions are the first-line treatment for overweight individuals (OW) and individuals with obesity (OB) with high-normal blood pressure (BP) or grade I hypertension, especially when at low-to-moderate cardiovascular risk (CVR). However, current guidelines do not specify the most effective dietary approach for optimising cardiovascular and metabolic outcomes in this population. This study aimed to compare the effects of a low-calorie, high-protein ketogenic diet (KD) vs. a low-calorie, low-sodium, and high-potassium Mediterranean diet (MD) on BP profiles assessed via ambulatory BP monitoring (ABPM), as well as on anthropometric measures, metabolic biomarkers, and body composition evaluated by bioelectrical impedance analysis (BIA). **Methods:** This prospective observational bicentric pilot study included 26 non-diabetic adult outpatients with central OW status or OB status (body mass index, BMI > 27 kg/m^2^) and high-normal BP (≥130/85 mmHg) or grade I hypertension (140–160/90–100 mmHg), based on office BP measurements. All participants had low-to-moderate CVR according to the second version of the systemic coronary risk estimation (SCORE2) and were selected and categorized as either KD (*n* = 15) or MD (*n* = 11). Comprehensive blood analysis, BIA, and ABPM were conducted at baseline and after three months. **Results:** At baseline, no significant differences were observed between the groups. Following three months of dietary intervention, both groups exhibited substantial reductions in body weight (KD: 98.6 ± 13.0 to 87.3 ± 13.4 kg; MD: 93.8 ± 17.7 to 86.1 ± 19.3 kg, *p* < 0.001) and waist circumference. Mean 24 h systolic BP (SBP) and diastolic BP (DBP) significantly declined in both groups (24 h mean SBP decreased from 125.0 ± 11.3 to 116.1 ± 8.5 mmHg (*p* = 0.003) and 24 h mean DBP decreased from 79.0 ± 8.4 to 73.7 ± 6.4 mmHg (*p* < 0.001)). Fat-free mass (FFM) increased, whereas fat mass (FM), blood lipid levels, and insulin concentrations decreased significantly. The ΔFM/ΔFFM correlates with ABP improvements. However, no significant between-group differences were detected at follow-up. **Conclusions:** The KD and the MD mediated weight loss and body composition changes, effectively improving bio-anthropometric and cardiovascular parameters in individuals with OW status or OB status and high BP. Although more extensive studies are warranted to elucidate potential long-term differences, our findings suggest the manner in which these two different popular dietary approaches may equally confer metabolic and cardiovascular benefits, emphasising the importance of weight and FM loss.

## 1. Introduction

The prevalence of overweight (OW) individuals and individuals with obesity (OB) is rising globally, particularly in Western countries. Excess accumulation of visceral adipose tissue is associated with various pathophysiological disturbances, ultimately contributing to elevated blood pressure (BP), dyslipidemia, impaired glucose homeostasis, and an increased risk of cardiovascular disease (CVD) [1]. Lifestyle modifications, including dietary interventions, represent the primary recommended approach for individuals with OW status or OB status who present with high-normal BP or grade I hypertension and low-to-moderate cardiovascular risk (CVR). The current guidelines advocate for comprehensive lifestyle interventions to reduce BP, including increased physical activity, sodium restriction, and adherence to a diet rich in vegetables, fruits, legumes, whole grains, fish, and unsaturated fatty acids, particularly olive oil. Additionally, low-fat dairy products are preferred over red meat and processed foods. At the same time, excessive alcohol intake should be minimised. Smoking cessation is strongly encouraged [2,3]. Notably, many of these dietary recommendations align with the key principles of the Mediterranean diet (MD), which has been extensively associated with a reduced incidence of cardiovascular events and all-cause mortality [4,5]. However, current guidelines do not specify a particular dietary regimen.

Recently, there has been increasing interest, among both the scientific community and patients, in ketogenic diets (KD), which have been proposed for weight and metabolic syndrome (MetS) management, given the hypothesis that the high lipid and protein content, combined with a low carbohydrate intake, may confer more significant metabolic and cardiovascular benefits, compared to other hypocaloric diets [6]. Conversely, KDs are often characterised by a high intake of sodium and saturated fatty acids, which may have detrimental effects, particularly in individuals who have OW status or OB status and elevated BP or even hypertension.

This study compared the effects of a hypocaloric, high-protein KD based on commonly available natural foods with those of a hypocaloric, low-sodium, high-potassium MD in individuals with OW status or OB status and high-normal BP or grade I hypertension who were classified as having low-to-moderate CVR. The primary endpoint was the change in 24 h mean systolic BP (SBP) and diastolic BP (DBP), assessed through ambulatory BP monitoring (ABPM) at baseline and after three months of dietary intervention. Secondary endpoints included changes in daytime and night-time mean SBP and DBP (as measured by ABPM), body weight, key anthropometric markers, biochemical parameters, body composition assessed via bioelectrical impedance analysis (BIA), and metabolic profile.

## 2. Materials and Methods

### 2.1. Study Design and Population

A prospective, observational, bicentric, open-label, and non-controlled pilot study was conducted on twenty-six consecutive outpatients referred to the European Hypertension Society (ESH) Excellence Centre, Istituto di Ricerca e Cura a Carattere Scientifico (IRCCS) Istituto Nazionale di Ricerca e Cura Anziani (INRCA), Ancona, Italy, and the Endocrinology and Metabolic Disease Clinic, Azienda Ospedaliero-Universitaria delle Marche, Ancona, Italy, between February 2023 and November 2024.

The inclusion criteria encompassed individuals aged ≥18 years and <80 years, with central OW or OB status, as defined by a body mass index (BMI) of 27–39.9 kg/m^2^ and a waist circumference (WC) > 102 cm in men and >88 cm in women. Participants presented with high-normal BP, characterised by office BP ≥ 130 and/or 85 mmHg, or grade I hypertension, defined as office BP ≥ 140 and/or 90 mmHg, but all were <160 and/or 100 mmHg, confirmed via ABPM, and were not undergoing antihypertensive treatment. Low-to-moderate CVR was confirmed using the Systematic Coronary Risk Estimation (SCORE2) by the validated web app https://www.humtelemed.it/ (accessed on 17 May 2025) [7]. Participants were required to be clinically eligible for either the MD or the KD and able to follow an unrestricted diet at enrollment. The exclusion criteria comprised individuals following a specific diet at baseline, those on antihypertensive therapy, or those diagnosed with metabolic or systemic diseases such as diabetes mellitus (DM), stage 3 chronic kidney disease (CKD) with an estimated glomerular filtration rate (eGFR) < 45 mL/min/1.73 m^2^ based on the CKD-EPI creatinine equation, oncologic disease, severe hypercholesterolemia (defined as low-density lipoprotein cholesterol (LDL-C) ≥ 190 mg/dL), severe hypertriglyceridemia (triglycerides or TG ≥ 400 mg/dL), hepatic cirrhosis or known heart disease. Additionally, patients unable to adhere to lifestyle recommendations and attend follow-up assessments and those requiring antihypertensive treatment during follow-up were excluded from the study.

Participants did not initiate pharmacological antihypertensive therapy but were assigned a three-month dietary intervention, adhering to the 2023 ESH hypertension guidelines [2] and Good Clinical Practice (GCP). All participants provided informed consent, and the study received ethical approval from the local institutional ethics committee (CE INRCA, Ancona, Italy; Approval Code: SC/14/443; Approval Date: 24 July 2014). The study was conducted in compliance with the principles of the Declaration of Helsinki and subsequent amendments.

### 2.2. Office and Ambulatory Blood Pressure Measurement

Office BP measurements were conducted following standard protocols. Three readings were taken at 1 to 2 min intervals, and the mean of the last two values was recorded. Participants were classified as having high-normal BP or grade I hypertension.

ABPM was performed using the Spacelabs model 90207 and 90217 devices with appropriately sized cuffs (Spacelabs Healthcare, Redmond, WA, USA). The higher average office BP value between values recorded for different arms was used for the analyses and to place the ABPM, thus avoiding errors due to interarm BP differences [8]. Measurements were recorded at 15 min intervals during the day (6:00 AM–10:00 PM) and every 20 min at night (10:00 PM–6:00 AM). The device provided mean BP values for each 24 h, daytime, and night-time period, along with an assessment of the night-to-day ratio, expressed by the ratio of mean night-time SBP to mean daytime SBP as a percentage, and an assessment of the dipping status (a nocturnal SBP reduction > 10% relative to daytime SBP was defined as “dipper” status). The definitions of “day” and “night” periods were based on the most common answers to a questionnaire in which patients were asked about their sleeping behaviour. The minimum quality criteria for a satisfactory ABPM recording were based on the published recommendations [9]. Participants also maintained a diary documenting daily activities (e.g., work, meals, and exercise) and sleep duration.

### 2.3. Dietary Intervention

A dietitian conducted a comprehensive nutritional anamnesis, and participants were assigned to either the KD or the MD based on their nutritional profile and personal preferences, as per good routine clinical practice. Given the nature of the study, neither the participants nor the dietitian were blinded to diet allocation; however, physicians and the statistician analysing the data remained blinded to group assignments.

The average macronutrient composition of the KD was 1300 ± 100 kcal/day (adjusted based on individual energy requirements), with carbohydrates constituting about 10–15% of total caloric intake (restricted to <50 g/day), fats 55–60% and proteins 25–30% (≥1.2 g/kg of ideal body weight per day). Sodium intake was, on average, ≥5 g per day but lower than 8 g/day, while potassium intake was <4 g/day.

The MD regimen provided 1300 ± 100 kcal/day, with carbohydrates accounting for 40–50% of total caloric intake (≥50 g/day), fats 35%, and proteins 15–25% (0.9 g/kg of ideal body weight per day). Sodium intake was maintained at <5 g/day, and potassium intake was set at 4 g/day.

Neither multivitamin nor multimineral supplementation nor any other supplement was provided, as both dietary regimens were designed to meet nutritional requirements. Adherence to the assigned dietary intervention was assessed monthly through in-person evaluations and regularly through telephone consultations with the dietitian [10]. Participants were also advised to increase physical activity (moderate endurance exercises for at least 30 min on five or more days per week) as suggested by the relevant guidelines [2]; however, no structured exercise program was implemented.

The physical activity was estimated by calculating the Physical Activity Level (PAL), based on the comprehensive nutritional anamnesis, to estimate energy requirements. PAL is the result of the ratio between total energy expenditure (TEE) and basal metabolic rate (BMR). BMR is estimated by equations based on parameters such as body weight, age, and gender (for example, the Harris–Benedict equation). According to IOM (Institute of Medicine, 2005), the PAL can classify the subjects into four categories: sedentary lifestyles, ranging between 1.00 and 1.39; low-activity lifestyles, ranging between 1.40 and 1.59; active lifestyles, ranging between 1.60 and 1.89; and very active lifestyles, ranging between 1.9 and 2.50 [11].

### 2.4. Baseline and Follow-Up Assessments

Anthropometric parameters, including height, body weight, and waist circumference, were recorded at baseline and follow-up. Height (meters) was measured without shoes, using a stadiometer, to the nearest 0.1 cm. Body weight (kg) was assessed with minimal clothing and no shoes, using a calibrated scale, to the nearest 0.1 kg. BMI was calculated as weight (kg) divided by height squared (m^2^). WC (cm) was measured in the horizontal plane at the midpoint between the lowest rib and the iliac crest, recorded to the nearest 0.1 cm at the end of a normal expiration.

Body composition was evaluated using bioelectrical impedance analysis (BIA) with the Imp DF50 Body Composition Analysis Device (ImpediMed Inc. 5900 Pasteur Ct.Ste. 125 Carlsbad, CA 92008 United States of America), measuring fat mass (FM, kg and %), fat-free mass (FFM, kg and %), total body water (TBW, litres and %), extracellular water (ECW, litres and %), intracellular water (ICW, litres and %), and phase angle (degrees).

Fasting blood samples were collected to analyse total cholesterol (TC), high-density lipoprotein cholesterol (HDL-C), TG, lipoprotein “a” [Lp(a)], apolipoprotein B (ApoB), uric acid (UA), fasting glucose, insulin, glycosylated haemoglobin (HbA1c), creatinine, urea, sodium, potassium, high-sensitivity C-reactive protein (HS-CRP), and thyroid hormones. LDL-C was estimated using the modified Friedewald equation proposed by Martin [12]. Non-HDL-C was calculated as TC minus HDL-C. Insulin resistance (IR) was assessed using the Homeostasis Model Assessment of IR (HOMA-IR): HOMA-IR = (fasting glucose [mg/dL] × fasting insulin [μU/mL])/405. Urinary biomarkers were collected, including microalbuminuria and 24 h sodium and potassium excretion. Participants collected 24 h urine samples from the first morning void to 7:00 AM the next day.

### 2.5. Statistical Analysis

Data were analysed using the Statistical Package for Social Sciences (SPSS Inc., Chicago, IL, USA) version 21. Normality was assessed for continuous variables, which were reported as mean ± standard deviation (SD) or median and interquartile range (IQR) if skewed. Categorical variables were expressed as frequencies and percentages. Pearson’s and Spearman’s tests expressed the correlation between variables. Group differences were analysed using the independent *t*-test, Mann–Whitney test, and χ^2^ test. Paired sample *t*-tests and Wilcoxon Signed Rank tests were employed for within-group comparisons between baseline and follow-up. Statistical significance was set at *p* < 0.05.

## 3. Results

### 3.1. Study Population

A total of 31 Caucasian individuals with central obesity were enrolled in the study. Five participants discontinued their participation prior to the completion of the study due to non-adherence to dietary protocols or the initiation of antihypertensive pharmacotherapy. The final analysis included 26 participants: 15 assigned to the ketogenic diet (KD) group and 11 to the Mediterranean diet (MD) group. Baseline and follow-up characteristics are presented in Table 1. The study’s population was predominantly middle-aged males, with anthropometric measurements indicative of central OB status. Baseline office and ABPM values were consistent with high-normal BP or grade I hypertension. There were no significant differences in baseline characteristics between the KD and MD groups (Supplementary Table S1).

### 3.2. Changes Following Dietary Intervention in the Overall Population

**Figure 1:**
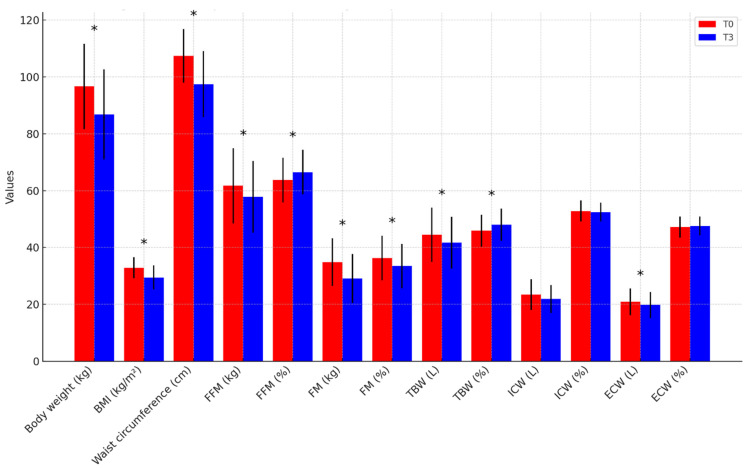
**Anthropometric and body composition parameters from baseline to follow-up in the overall population.** * *p*-value < 0.05. BMI = Body Mass Index, FFM = Free Fat Mass, FM = Fat Mass, TBW = Total Body Water, ICW = Intra-Cellular Water, ECW = Extra-Cellular Water.

**Figure 2:**
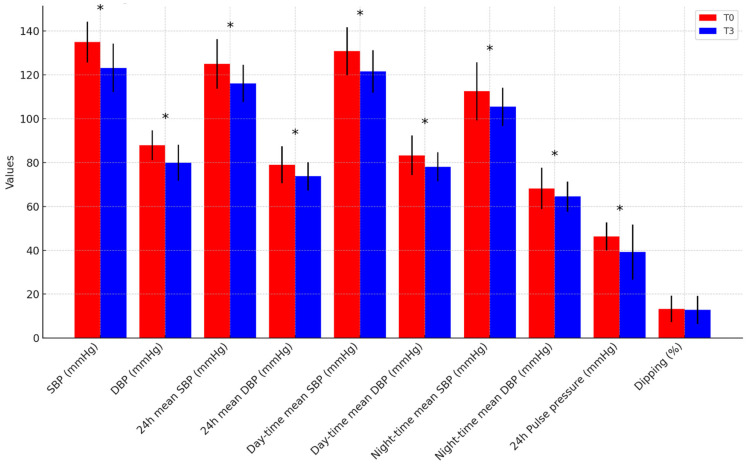
**Blood pressure parameters from baseline to follow-up in the overall population.** * *p*-value < 0.05. SBP = Systolic Blood Pressure, DBP = Diastolic Blood Pressure.

**Figure 3:**
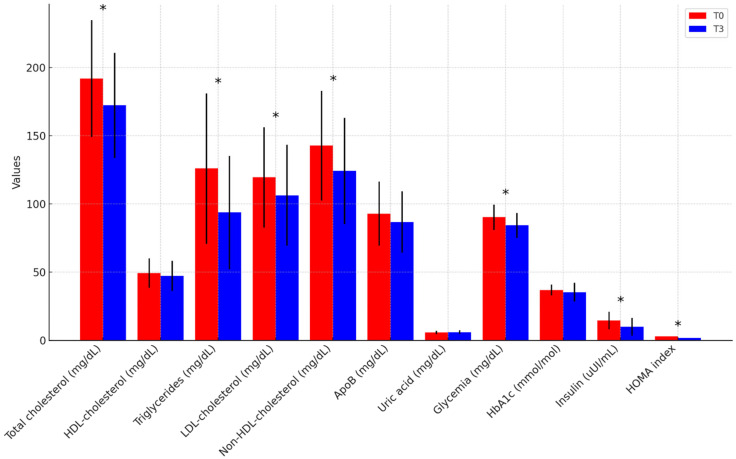
**Metabolic parameters from baseline to follow-up in the overall population.** * *p*-value < 0.05. HDL = High-Density Lipoprotein, LDL = Low-Density Lipoprotein, ApoB = Apolipoprotein B, HbA1c = Glycated Haemoglobin, HOMA = Homeostatic Model Assessment.

At the end of the 3-month intervention period, the overall cohort demonstrated statistically significant improvements in anthropometric indices, body composition, blood pressure, and metabolic parameters, independent of diet type (Supplementary Table S2). Regarding anthropometrics and body composition (Figure 1), weight decreased from 96.6 ± 15.0 to 86.8 ± 15.8 kg (*p* < 0.001), WC decreased from 107.4 ± 9.4 to 97.4 ± 11.6 cm (*p* < 0.001), and BMI decreased from 32.9 ± 3.7 to 29.5 ± 4.2 kg/m^2^ (*p* < 0.001); FM significantly decreased in absolute terms (34.9 ± 8.4 to 29.1 ± 8.6 kg, *p* < 0.001) and as a percentage (36.3 ± 7.8% to 33.5 ± 7.8%, *p* < 0.001); in contrast, FFM decreased in absolute values (61.7 ± 13.2 to 57.8 ± 12.6 kg, *p* < 0.001) but increased proportionally (63.7 ± 7.8% to 66.5 ± 7.8%, *p* < 0.001). Concerning BP profile (Figure 2), office SBP decreased from 134.9 ± 9.3 to 123.2 ± 11.1 mmHg (*p* < 0.001), and office DBP decreased from 87.9 ± 6.8 to 80.0 ± 8.2 mmHg (*p* < 0.001); 24 h mean SBP decreased from 125.0 ± 11.3 to 116.1 ± 8.5 mmHg (*p* = 0.003) and 24 h mean DBP decreased from 79.0 ± 8.4 to 73.7 ± 6.4 mmHg (*p* < 0.001); daytime mean SBP decreased from 130.8 ± 10.9 to 121.5 ± 9.7 mmHg (*p* = 0.003) and daytime mean DBP decreased from 83.3 ± 9.0 to 78.1 ± 6.6 mmHg (*p* = 0.001); night-time mean SBP decreased from 112.5 ± 13.3 to 105.4 ± 8.7 mmHg (*p* = 0.011) and night-time mean DBP decreased from 68.2 ± 9.4 to 64.5 ± 6.8 mmHg (*p* = 0.017). The proportion of participants with a dipper BP pattern remained stable from baseline to follow-up (65.4% vs. 66.7%, *p* > 0.05). As for the metabolic parameters (Figure 3), TC decreased from 192.1 ± 42.9 to 172.3 ± 38.7 mg/dL (*p* = 0.012), TG decreased from 126.0 ± 55.2 to 93.7 ± 41.6 mg/dL (*p* = 0.016), LDL-C decreased from 119.5 ± 36.7 to 106.3 ± 37.0 mg/dL (*p* = 0.046), and non-HDL-C decreased from 142.8 ± 40.3 to 124.2 ± 39.0 mg/dL (*p* = 0.014). Basal fasting glycemia decreased from 90.2 ± 9.4 to 84.2 ± 9.2 mg/dL (*p* = 0.002), insulin levels decreased from 14.4 ± 6.4 to 9.8 ± 6.4 μUI/mL (*p* = 0.002), and, consequently, HOMA-IR significantly improved from 2.89 (2.06–4.51) to 1.73 (1.15–2.76) (*p* = 0.001). No significant changes were observed in HDL-C, ApoB, HbA1c, or serum uric acid (UA) levels (*p* > 0.05). Inflammatory and renal function markers, including serum creatinine, urea, potassium, and high-sensitivity C-reactive protein (hs-CRP), remained unchanged. After three months, 24 h sodium excretion decreased from 184.4 ± 79.7 to 145.4 ± 77.3 mEq/24 h (*p* = 0.102), while 24 h potassium excretion increased from 60.4 ± 28.3 to 66.8 ± 31.7 mEq/24 h (*p* = 0.498), though neither change reached statistical significance.

All participants were sedentary at baseline, according to physical activity level (PAL) classifications. At follow-up, only 10 participants (38.5%) transitioned to the “low activity” category, indicating minimal change in physical activity status during the intervention. No significant correlations were observed between changes in anthropometric or metabolic parameters and ambulatory BP. However, a moderate, statistically significant correlation was found between the FM-to-FFM change ratio (ΔFM/ΔFFM) and reductions in mean SBP. Specifically, correlation coefficients were r = 0.569 for 24 h SBP, r = 0.619 for daytime SBP, and r = 0.458 for night-time SBP (Supplementary Table S3).

### 3.3. Comparative Effects of the KD and MD

**Figure 4:**
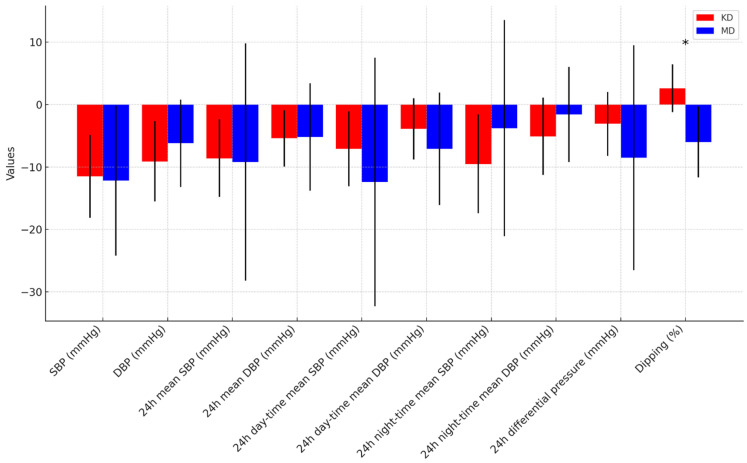
Change in blood pressure parameters between follow-up and baseline, according to diet. * *p*-value < 0.05. SBP = Systolic Blood Pressure, DBP = Diastolic Blood Pressure.

Both the KD and the MD groups showed significant within-group reductions in body weight, BMI, FM, office BP, and ambulatory BP parameters (Figure 4). No statistically significant differences were observed between the diets in their effects on anthropometric, cardiovascular, or metabolic markers.

The only exception was the night-time BP dipping percentage, which significantly diverged between groups. In the KD group, the night-to-day SBP ratio increased from 12.4 ± 6.3% to 15.0 ± 4.9%, whereas in the MD group, it decreased from 16.3 ± 5.8% to 10.3 ± 7.3%. A between-group comparison revealed a significant difference (mean change: +2.6 ± 3.8% vs. −6.0 ± 5.7%, *p* < 0.001)

Table 1 summarises the characteristics of the study population at baseline and follow-up, according to diet, and Table 2 reports the mean differences between baseline and follow-up, according to diet.

## 4. Discussion

This pilot study was conducted on a population of non-diabetic adult outpatients presenting with central OW status or OB status, elevated BP up to grade I hypertension, and low-to-moderate CVR. The primary objective was to evaluate the impacts of two dietary interventions—the KD and the MD—on BP profiles assessed via ABPM, anthropometric measurements, metabolic parameters, and body composition.

The preliminary findings demonstrated the beneficial effects of dietary intervention on body weight, WC, metabolic parameters (particularly the HOMA index and lipid profile), and body composition, regardless of the specific diet prescribed. Both the KD and the MD similarly improved overall ABP profiles, and no significant differences were observed between the two dietary regimens concerning their effects on the analysed parameters. These results reinforce the hypothesis that adherence to hypocaloric diets under dietitian supervision, leading to substantial weight reduction, is pivotal in mitigating metabolic disturbances closely linked to increased BP.

It is well-established that a mean reduction of 4.4 mmHg in SBP and 3.6 mmHg in DBP can be achieved with an average weight loss of approximately 5 kg [13]. Accordingly, hypertension guidelines consistently recommend weight reduction as the most effective lifestyle intervention for lowering BP in individuals with OW status or OB status and high-normal BP or grade I hypertension, even before the initiation of antihypertensive pharmacological treatment [2,3]. For patients with low CVR, BP-lowering medication should be initiated after 3–6 months if lifestyle interventions alone are insufficient. Weight reduction, achieved through a multidisciplinary approach including dietary modifications and increased physical activity, remains a fundamental recommendation with the highest level of evidence [14,15] and is based on potential weight loss-related BP-lowering mechanisms likely associated with caloric and salt restriction and haemodynamic effects.

Innovative therapeutic approaches based on incretins may provide a valuable alternative in clinical contexts where continuous dietitian monitoring is not feasible. Among these, semaglutide has demonstrated significant efficacy in non-diabetic patients, as evidenced by the STEP 1 Trial [16], which reported the first documentation of BP reduction. Similarly, tirzepatide has shown promising outcomes, particularly in BP profiles confirmed through ABPM, as highlighted in the SURMOUNT-1 ABPM substudy [17]. Post hoc analyses from the same SURMOUNT-1 trial indicate that weight loss may account for approximately 68% of the reduction in SBP and 71% of the reduction in DBP [18]. This suggests that BP-lowering mechanisms may be related to caloric and salt restriction and reductions in humoral and sympathetic hemodynamic effects. Leveraging the capabilities of the BIA, the present findings—derived from correlation analyses—indicate that while absolute changes in FM and FFM did not independently exhibit statistically significant associations with improvements in ABP profiles, the relative change between these parameters, as represented by the ΔFM/ΔFFM ratio, demonstrated a significant relationship with reductions in mean SBP. Moderate correlations between the ΔFM/ΔFFM ratio and improvements in 24 h, daytime, and night-time SBP (r = 0.458 to 0.619) suggest that a favourable alteration in body composition—specifically, a more pronounced reduction in FM relative to increases in FFM—may play a meaningful role in enhancing blood pressure regulation.

These results, despite being preliminary, underscore the potential clinical significance of pursuing overall weight loss and qualitative changes in body composition to optimise cardiovascular health outcomes. The relevance of relative changes in body composition to ABP regulation is further supported by findings from a study employing an inverse design. In this investigation, the researchers examined the effects of experimentally induced weight gain on blood pressure among healthy individuals, with particular attention to the relationship between ABP changes and fat distribution. Over eight weeks, participants who experienced weight gain exhibited increased FM, especially in visceral regions, as confirmed by imaging modalities, and concomitantly displayed elevated 24 h BP. In contrast, no significant changes were observed in the control group. Notably, increases in mean ABP were explicitly associated with increases in visceral FM. These findings suggest that even modest weight gain can adversely affect BP, with visceral adiposity playing a pivotal role in this physiological response [19].

Regarding specific dietary interventions, there is broad consensus as to implementing sodium restriction, yet no agreement has been reached on potassium supplementation [20]. Although potassium intake may enhance natriuresis by deactivating the renal distal tubular sodium-chloride cotransporter (NCC), the direct impact on BP remains uncertain [21]. Notably, the dietary approach to stop hypertension (DASH) diet, which is rich in potassium, low-fat dairy, whole grains, and lean protein, has been associated with a reduction of approximately 11 mmHg in hypertensive individuals and 3 mmHg in normotensive adults [22]. Similarly, the MD, which shares some features with the DASH diet but includes moderate alcohol intake, demonstrates BP-lowering effects and is recommended by European hypertension guidelines [2]. Furthermore, a recent intersociety guideline document addressing the application of the MD across various clinical settings—including cardiovascular prevention, weight management, and metabolic control—has been developed and approved by the Italian National Institute of Health (Istituto Superiore di Sanità, ISS). Within this document, the MD received strong recommendations for its efficacy in reducing the incidence of DM2, cardiovascular mortality, ischemic stroke, atrial fibrillation, and peripheral artery disease [23]. On the other hand, the KD has recently gained attention as a nutritional strategy for weight management. However, its effects on BP remain underexplored, with inconclusive findings primarily due to heterogeneity in study designs and dietary composition [24]. Most evidence pertains to very-low-calorie ketogenic diets (VLCKD), which differ significantly from the standard KD used in the present study [25]. Notably, no randomized controlled trial (RCT) has directly compared a standardised KD with the MD in terms of BP modulation among individuals with OW status or OB status and low-grade hypertension.

Although no difference emerged in the mean ABPs, our preliminary findings suggest that the KD has a more pronounced effect on nocturnal BP dipping than does the MD.

The KD, compared to other nutritional strategies for weight reduction, may be associated with distinctive beneficial effects on the central nervous system, including improvements in sleep quality [26,27,28] and a decrease in sympathetic nervous system activity, which may play a critical role in preserving the physiological nocturnal decline in BP after adherence to a low-calorie KD, as compared to a fed state, and in contrast to a standard low-calorie diet, further supporting the putative effects attributed to ketone bodies [29,30]. Nevertheless, most available evidence is derived from studies employing VLCKDs. The precise role of ketogenic diets—or ketone supplementation—in improving nocturnal BP control and promoting the expected nocturnal dipping pattern remains incompletely elucidated. Ongoing clinical trials (e.g., NCT05888506) are currently investigating the hypothesis that acute ketone supplementation may reduce night-time BP and enhance dipping patterns, compared to placebo. However, these preliminary results warrant further investigation, which should consider specific possible influencing factors (e.g., obstructive sleep apnea syndrome).

Consistent with the previous research, both the KD and the MD significantly improved metabolic parameters, including fasting insulin, HbA1c, HOMA-IR, and lipid profiles [31]. A meta-analysis demonstrated that the KD significantly reduces FM when carbohydrate intake is restricted to ≤50 g/day [32]. Although the present study lacks long-term follow-up data, the literature supports the association of the MD with sustained weight loss and long-term metabolic benefits [33]. However, findings as to the KD’s long-term effects remain less consistent, with some studies failing to demonstrate significant improvements in weight and metabolic parameters [34].

Both dietary interventions were well-tolerated, with a high percentage of persistence, and no adverse effects or significant changes in renal function markers were reported [35].

### Strengths and Limitations

Using ABPM to evaluate BP profiles at baseline and follow-up allows for greater accuracy in assessing BP changes [36] and represents the main strength of our study. ABPM has a greater reproducibility than office BP and provides a closer prediction of hypertension-mediated organ damage, CV outcomes, and mortality [2]. Another strength of our study lies in the comprehensive range of analysed parameters, including complete BP measurements, anthropometric assessments, an extensive array of metabolic biomarkers, and detailed body composition changes, thanks to the BIA. This approach enables the accurate and thorough characterisation of the study population.

The primary limitations of this study are the small sample size, the absence of a control group, and the short follow-up. Given the cardiometabolic risk profile of the participants, we believed that the use of a “control” group might be considered ethically inappropriate in a context of a prospective cohort study. In the study design phase, we did not choose to allocate patients to specific dietary regimens through randomization. Due to the intrusive nature of nutritional modifications, allowing participants to select a diet according to their preferences was likely to enhance adherence. This was also an attempt to keep the study as close as possible to what would happen in real, daily clinical practice. Although there was no randomization, the KD and MD populations were well balanced concerning the parameters of interest (anthropometric and bioimpedance measurements, laboratory metabolic parameters, and baseline BP parameters).

Moreover, although patients were closely monitored during the 3-month follow-up, the study protocol did not incorporate precise and objective tools/questionnaires for assessing adherence to dietary interventions and physical activity during follow-up. Nonetheless, to objectively verify dietary compliance, 24 h urinary sodium and potassium excretion levels were measured as surrogate markers, despite this method’s limited reflection of real-world clinical practice. The essential coherence of all the parameters examined, trending towards the improvement of a weight loss of about 10 kg in 3 months, testifies to the good adherence to the dietary intervention of the enrolled patients. 

Therefore, this paper was configured to be a pilot study with preliminary data, since there is no direct comparison between these two diets in the literature. Consequently, the restricted generalizability of the findings necessitates further research with larger cohorts, a randomized controlled design, and extended follow-up to validate the findings and assess the durability of these effects. However, the stringent inclusion criteria enabled the investigation of the impacts of dietary lifestyle interventions within a real-life population of patients who exhibited the most relevant clinical indications. This approach enhances the study’s internal validity and ensures that the findings apply to the individuals who would most likely benefit from such interventions.

## 5. Conclusions

Our preliminary results, although obtained in a small population of carefully studied patients, add novel information in this field because the weight loss obtained with a low-calorie diet, either the KD with regular foods or an MD, is effective and safe in the short-to-medium term in patients with OW/OB and elevated BP or grade 1 hypertension. Our study, therefore, underscores the significance of weight loss and the associated improvement in body composition (especially FM reduction over FFM gain), irrespective of the method employed to achieve it, as it confers comprehensive benefits across the entire cardiometabolic profile. If these data are confirmed in clinical trials with broader populations, future guidelines and recommendations on treating elevated BP and hypertension management can also consider this evidence on lifestyle.

## Figures and Tables

**Table 1:** Characteristics of the study population at baseline and follow-up, according to diet.

Parameters	KD (*n* = 15)		MD (*n* = 11)		*p*-Value *
	T0	T3	T0	T3	
**Age (years)**	41.7 ± 11.4	-	52.0 ± 10.2	-	-
**Sex (%, male)**	73.3%	-	54.5%	-	-
**Body weight (kg)**	98.6 ± 13.0	87.3 ± 13.4	93.8 ± 17.7	86.1 ± 19.3	*p* = 0.849
**BMI (kg/m^2^)**	33.5 ± 4.0	29.6 ± 4.2	32.0 ± 3.4	29.4 ± 4.2	*p* = 0.874
**Waist circumference (cm)**	107.2 ± 7.2	95.4 ± 9.4	107.8 ± 12.1	100.0 ± 13.4	*p* = 0.313
**FFM (kg)**	63.6 ± 11.3	59.1 ± 10.4	59.0 ± 15.6	56.1 ± 15.5	*p* = 0.557
**FFM (%)**	64.6 ± 8.0	67.8 ± 8.2	62.4 ± 7.7	64.8 ± 7.2	*p* = 0.340
**FM (kg)**	34.9 ± 9.3	28.4 ± 9.2	34.7 ± 7.4	30.0 ± 8.0	*p* = 0.638
**FM (%)**	35.4 ± 8.0	32.2 ± 8.2	37.6 ± 7.7	35.2 ± 7.2	*p* = 0.340
**TBW (L)**	45.9 ± 8.1	42.6 ± 7.5	42.5 ± 11.2	40.5 ± 11.1	*p* = 0.557
**TBW (%)**	46.5 ± 5.7	48.9 ± 5.9	45.0 ± 5.6	46.7 ± 5.2	*p* = 0.341
**ICW (L)**	24.8 ± 4.9	22.8 ± 4.3	21.7 ± 5.8	20.5 ± 5.5	*p* = 0.236
**ICW (%)**	54.0 ± 3.8	53.6 ± 3.1	51.2 ± 2.8	50.8 ± 3.1	*p* = 0.029
**ECW (L)**	21.1 ± 4.1	19.8 ± 3.6	20.8 ± 5.7	19.9 ± 5.9	*p* = 0.926
**ECW (%)**	46.0 ± 3.8	46.4 ± 3.1	48.8 ± 2.8	49.2 ± 3.1	*p* = 0.029
**SBP (mmHg)**	134.2 ± 8.4	122.7 ± 7.2	135.9 ± 10.8	123.7 ± 15.4	*p* = 0.824
**DBP (mmHg)**	88.6 ± 6.6	79.5 ± 6.8	86.9 ± 7.3	80.6 ± 10.1	*p* = 0.741
**24 h mean SBP (mmHg)**	127.3 ± 9.9	118.3 ± 7.4	123.9 ± 13.2	113.1 ± 9.5	*p* = 0.146
**24 h mean DBP (mmHg)**	81.2 ± 8.0	75.6 ± 5.8	77.2 ± 8.4	71.0 ± 6.6	*p* = 0.085
**Daytime mean SBP (mmHg)**	131.8 ± 9.4	124.6 ± 7.0	131.3 ± 13.2	117.3 ± 11.6	*p* = 0.069
**Daytime mean DBP (mmHg)**	84.3 ± 8.6	80.3 ± 5.0	83.2 ± 9.6	75.0 ± 7.5	*p* = 0.050
**Night-time mean SBP (mmHg)**	116.2 ± 12.8	105.6 ± 9.0	110.1 ± 13.9	104.7 ± 8.6	*p* = 0.756
**Night-time mean DBP (mmHg)**	71.2 ± 10.4	65.6 ± 7.4	65.5 ± 6.7	63.1 ± 5.8	*p* = 0.391
**24 h pulse pressure (mmHg)**	46.1 ± 5.3	39.9 ± 11.8	46.7 ± 8.0	38.3 ± 14.2	*p* = 0.758
**Dipping (%)**	12.4 ± 6.3	15 ± 4.9	16.3 ± 5.8	10.3 ±7.3	*p* = 0.074
**Total cholesterol (mg/dL)**	192.2 ± 44.1	170.6 ± 40.8	191.9 ± 43.5	174.7 ± 37.4	*p* = 0.794
**HDL-cholesterol (mg/dL)**	47.9 ± 10.0	47.2 ± 12.1	51.2 ± 12.1	47.2 ± 10.2	*p* = 0.997
**Triglycerides (mg/dL)**	129.7 ± 66.3	86.1 ± 47.3	120.8 ± 37.3	104.0 ± 31.6	*p* = 0.289
**LDL-cholesterol (mg/dL)**	120.5 ± 36.8	105.7 ± 38.7	118.1 ± 38.4	107.2 ± 36.2	*p* = 0.925
**Non-HDL-cholesterol (mg/dL)**	144.3 ± 41.8	122.3 ± 40.1	140.7 ± 40.1	126.7 ± 39.1	*p* = 0.783
**ApoB (mg/dL)**	92.9 ± 21.4	85.9 ± 19.9	92.6 ± 27.0	89.2 ± 25.7	*p* = 0.714
**Uric acid (mg/dL)**	5.8 ± 0.8	6.2 ± 1.3	5.2 ± 1.4	5.2 ± 1.3	*p* = 0.063
**Glycemia (mg/dL)**	87.7 ± 8.9	83.3 ± 10.3	93.5 ± 9.3	85.3 ± 7.8	*p* = 0.605
**HbA1c** **(mmol/mol)**	35.4 ± 3.8	32.7 ± 8.1	38.8 ± 3.2	38.6 ± 2.4	*p* = 0.028
**Insulin (uUI/mL)**	14.8 ± 7.4	9.8 ± 7.4	14.0 ± 5.0	9.7 ± 5.1	*p* = 0.971
**Creatinine (mg/dL)**	0.8 ± 0.2	0.8 ± 0.2	0.9 ± 0.2	0.9 ± 0.2	*p* = 0.402
**Urea (mg/dL)**	34.1 ± 7.3	35.6 ± 10.6	37.2 ± 7.5	33.4 ± 8.9	*p* = 0.576
**Sodium (mEq/L)**	139.9 ± 1.9	140.5 ± 2.2	140.5 ± 1.9	142.1 ± 3.1	*p* = 0.145
**Potassium (mEq/L)**	4.2 ± 0.6	4.4 ± 0.5	4.5 ± 0.3	4.4 ± 0.3	*p* = 0.699
**HS-CRP** **(mg/dL)**	0.82 (0.52–3.31)	0.79 (0.32–2.11)	3.29 (1.71–5.49)	2.76 (1.43–6.83)	*p* = 0.081
**24 h Na excretion (mEq/24 h)**	198.1 ± 89.6	149.4 ± 84.0	169.0 ± 61.3	140.0 ± 72.0	*p* = 0.790
**24 h K excretion (mEq/24 h)**	58.3 ± 26.0	70.5 ± 36.9	67.7 ± 29.6	61.8 ± 24.1	*p* = 0.548
**HOMA index**	2.86 (1.69–3.75)	1.79 (1.01–2.88)	2.90 (2.09–4.36)	1.71 (1.38–3.00)	*p* = 0.967
**Albuminuria/Creatininuria (mg/g)**	7.00 (2.35–12)	-	5.6 (4.10–14.70)	-	*p* = 0.849
**24 h cortisol excretion (mcg/24 h)**	164.6 (97.4–345)	-	120 (41.4–272.1)	-	*p* = 0.874

* *p*-value is for the difference between KD and MD at T3. BMI = Body Mass Index, FFM = Free Fat Mass, FM = Fat Mass, TBW = Total Body Water, ICW = Intra-Cellular Water, ECW = Extra-Cellular Water, SBP = Systolic Blood Pressure, DBP = Diastolic Blood Pressure, HDL = High-Density Lipoprotein, LDL = Low-Density Lipoprotein, ApoB = Apolipoprotein B, HbA1c = Glycated Haemoglobin, HS-CRP = High-Sensitivity C-Reactive Protein, HOMA = Homeostatic Model Assessment.

**Table 2:** Mean differences between follow-up and baseline, according to diet.

Parameter	Δ T3-T0 KD	Δ T3-T0 MD	*p*-Value
**Body weight (kg)**	−11.3 ± 4.2	−7.7 ± 5.1	*p* = 0.058
**BMI (kg/m^2^)**	−3.9 ± 1.4	−2.7 ± 1.8	*p* = 0.064
**Waist circumference (cm)**	−11.8 ± 4.1	−7.7 ± 7.0	*p* = 0.077
**FFM (kg)**	−4.5 ± 2.3	−2.9 ± 2.3	*p* = 0.083
**FFM (%)**	3.2 ± 1.7	2.4 ± 2.2	*p* = 0.250
**FM (kg)**	−6.6 ± 2.3	−4.7 ± 3.4	*p* = 0.107
**FM (%)**	−3.2 ± 1.6	−2.3 ± 2.1	*p* = 0.250
**TBW (L)**	−3.2 ± 1.7	−2.1 ± 1.7	*p* = 0.090
**TBW (%)**	2.3 ± 1.2	1.7 ± 1.6	*p* = 0.258
**ICW (L)**	−1.9 ± 1.7	−1.2 ± 0.9	*p* = 0.223
**ICW (%)**	−0.4 ± 3.2	−0.4 ± 1.1	*p* = 0.955
**ECW (L)**	−1.3 ± 1.8	−0.8 ± 1.1	*p* = 0.420
**ECW (%)**	0.4 ± 3.2	0.4 ± 1.1	*p* = 0.955
**SBP (mmHg)**	−11.5 ± 6.6	−12.2 ± 12.0	*p* = 0.852
**DBP (mmHg)**	−9.1 ± 6.4	−6.2 ± 7.0	*p* = 0.294
**24 h mean SBP (mmHg)**	−8.6 ± 6.2	−9.2 ± 19.0	*p* = 0.909
**24 h mean DBP (mmHg)**	−5.4 ± 4.5	−5.2 ± 8.6	*p* = 0.933
**24 h daytime mean SBP (mmHg)**	−7.1 ± 6.0	−12.4 ± 19.9	*p* = 0.352
**24 h daytime mean DBP (mmHg)**	−3.9 ± 4.9	−7.1 ± 9.0	*p* = 0.267
**24 h night-time mean SBP (mmHg)**	−9.5 ± 7.9	−3.8 ± 17.3	*p* = 0.287
**24 h night-time mean DBP (mmHg)**	−5.1 ± 6.2	−1.6 ± 7.6	*p* = 0.231
**24 h differential pressure (mmHg)**	−3.1 ± 5.1	−8.5 ± 18.0	*p* = 0.302
**Dipping (%)**	2.6 ± 3.8	−6.0 ± 5.7	*p* < 0.001
**Total cholesterol (mg/dL)**	−21.6 ± 45.5	−17.2 ± 22.3	*p* = 0.770
**HDL-cholesterol (mg/dL)**	−0.7 ± 8.0	−4.0 ± 6.8	*p* = 0.277
**Triglycerides (mg/dL)**	−43.6 ± 75.5	−16.8 ± 40.7	*p* = 0.298
**LDL-cholesterol (mg/dL)**	−14.9 ± 39.9	−11.0 ± 18.4	*p* = 0.769
**Non-HDL-cholesterol (mg/dL)**	−22.0 ± 43.4	−14.0 ± 22.8	*p* = 0.584
**ApoB (mg/dL)**	−8.1 ± 24.6	−3.5 ± 17.4	*p* = 0.604
**Uric acid (mg/dL)**	0.4 ± 0.9	0.0 ± 1.0	*p* = 0.319
**Glycemia (mg/dL)**	−4.3 ± 8.2	−8.3 ± 9.9	*p* = 0.277
**HbA1c** **(%)**	−0.3 ± 0.7	−0.0 ± 0.2	*p* = 0.338
**Insulin (µUI/mL)**	−4.9 ± 7.4	−4.3 ± 5.9	*p* = 0.813
**Creatinine (mg/dL)**	−0.0 ± 0.1	−0.0 ± 0.1	*p* = 0.810
**Urea (mg/dL)**	1.5 ± 6.8	−3.8 ± 6.0	*p* = 0.048
**Sodium (mEq/L)**	0.6 ± 2.2	1.6 ± 2.8	*p* = 0.301
**Potassium (mEq/L)**	0.2 ± 0.5	−0.1 ± 0.3	*p* = 0.081
**HS-CRP** **(mg/L)**	−1.7 ± 4.7	4.9 ± 13.6	*p* = 0.195
**24 h Na excretion (mEq/24 h)**	−42.6 ± 127.4	−34.3 ± 70.3	*p* = 0.863
**24 h K excretion (mEq/24 h)**	15.2 ± 49.4	−5.5 ± 28.0	*p* = 0.276
**HOMA index**	−1.1 ± 1.3	−1.1 ± 1.5	*p* = 0.993

BMI = Body Mass Index, FFM = Free Fat Mass, FM = Fat Mass, TBW = Total Body Water, ICW = Intra-Cellular Water, ECW = Extra-Cellular Water, SBP = Systolic Blood Pressure, DBP = Diastolic Blood Pressure, HDL = High-Density Lipoprotein, LDL = Low-Density Lipoprotein, ApoB = Apolipoprotein B, HbA1c = Glycated Haemoglobin, HS-CRP = High-Sensitivity C-Reactive Protein, HOMA = Homeostatic Model Assessment.

## Data Availability

The original contributions presented in this study are included in the article and the Supplementary Material. Further inquiries can be directed to the corresponding author.

## Supplementary Materials

The following supporting information can be downloaded at https://www.mdpi.com/article/10.3390/nu17101739/s1, Table S1: Characteristics of the overall study population and according to diet at baseline; Table S2: Characteristics of the overall study population at baseline and follow-up; Table S3: Correlations between the 24 h, daytime and night-time systolic blood pressure changes and the variation in anthropometric, body composition, and metabolic parameters.

## Abbreviations

The following abbreviations are used in this manuscript:

OW	Overweight
OB	Obesity
BP	Blood Pressure
KD	Ketogenic Diet
MD	Mediterranean Diet
ABPM	Ambulatory Blood Pressure Monitoring
BIA	Bioelectrical Impedance Analysis
BMI	Body Mass Index
SCORE2	Systematic Coronary Risk Estimation 2
FFM	Free Fat Mass
FM	Fat Mass
CVD	Cardiovascular Disease
SBP	Systolic Blood Pressure
DBP	Diastolic Blood Pressure
ESH	European Society of Hypertension
WC	Waist Circumference
DM	Diabetes Mellitus
CKD	Chronic Kidney Disease
eGFR	Estimated Glomerular Filtration Rate
LDL-C	Low-Density Lipoprotein Cholesterol
TG	Triglycerides
GCP	Good Clinical Practice
TBW	Total Body Water
ECW	Extra-Cellular Water
ICW	Intra-Cellular Water
TC	Total Cholesterol
HDL-C	High-Density Lipoprotein Cholesterol
ApoB	Apolipoprotein B
UA	Uric Acid
HbA1c	Glycosylated Haemoglobin
HS-CRP	High-Sensitivity C-Reactive Protein
IR	Insulin Resistance
HOMA-IR	Homeostasis Model Assessment of Insulin Resistance
SD	Standard Deviation
IQR	Interquartile Range
SPSS	Statistical Package for Social Sciences
NCC	Sodium-Chloride Cotransporter
DASH	Dietary Approach to Stop Hypertension
VLCKD	Very-Low-Calorie Ketogenic Diet
RCT	Randomized Controlled Trial
