# Supplementary material for: Low-Calorie, High-Protein Ketogenic Diet Versus Low-Calorie, Low-Sodium, and High-Potassium Mediterranean Diet in Overweight Patients and Patients with Obesity with High-Normal Blood Pressure or Grade I Hypertension: The Keto–Salt Pilot Study
Source: Nutrients. 2025 May 20;17(10):1739. doi: 10.3390/nu17101739 (PMC12114320; doi:10.3390/nu17101739)
Supplement: Supplementary file 1 [file nutrients-17-01739-s001.zip › nutrients-3620048-supplementary.pdf]

Table S1. Characteristics of the overall study population and according to diet at baseline

Parameter T0	Overall (n = 26)	KD (n = 15)	MD (n = 11)	p-value*
**Age (years)**	46 ± 11.9	41.7 ± 11.4	52.0 ± 10.2	p=0.070
**Sex (%, male)**	65.4%	73.3%	54.5%	p=0.419
**Body weight (kg)**	96.6 ± 15.0	98.6 ± 13.0	93.8 ± 17.7	p=0.427
**BMI (kg/m^2)**	32.9 ± 3.7	33.5 ± 4.0	32.0 ± 3.4	p=0.332
**Waist circumference (cm)**	107.4 ± 9.4	107.2 ± 7.2	107.8 ± 12.1	p=0.881
**FFM (kg)**	61.7 ± 13.2	63.6 ± 11.3	59.0 ± 15.6	p=0.385
**FFM (%)**	63.7 ± 7.8	64.6 ± 8.0	62.4 ± 7.7	p=0.502
**FM (kg)**	34.9 ± 8.4	34.9 ± 9.3	34.7 ± 7.4	p=0.947
**FM (%)**	36.3 ± 7.8	35.4 ± 8.0	37.6 ± 7.7	p=0.502
**TBW (L)**	44.5 ± 9.5	45.9 ± 8.1	42.5 ± 11.2	p=0.388
**TBW (%)**	45.9 ± 5.6	46.5 ± 5.7	45.0 ± 5.6	p=0.503
**ICW (L)**	23.5 ± 5.4	24.8 ± 4.9	21.7 ± 5.8	p=0.157
**ICW (%)**	52.8 ± 3.7	54.0 ± 3.8	51.2 ± 2.8	p=0.054
**ECW (L)**	20.9 ± 4.7	21.1 ± 4.1	20.8 ± 5.7	p=0.869
**ECW (%)**	47.2 ± 3.7	46.0 ± 3.8	48.8 ± 2.8	p=0.054
**SBP (mmHg)**	134.9 ± 9.3	134.2 ± 8.4	135.9 ± 10.8	p=0.656
**DBP (mmHg)**	87.9 ± 6.8	88.6 ± 6.6	86.9 ± 7.3	p=0.534
**24h mean SBP (mmHg)**	125 ± 11.3	127.3 ± 9.9	123.9 ± 13.2	p=0.465
**24h mean DBP (mmHg)**	79.0 ± 8.4	81.2 ± 8.0	77.2 ± 8.4	p=0.226
**24h day-time mean SBP (mmHg)**	130.8 ± 10.9	131.8 ± 9.4	131.3 ± 13.2	p=0.906
**24h day-time mean DBP (mmHg)**	83.3 ± 9.0	84.3 ± 8.6	83.2 ± 9.6	p=0.765
**24h night-time mean SBP (mmHg)**	112.5 ± 13.3	116.2 ± 12.8	110.1 ± 13.9	p=0.258
**24h night-time mean DBP (mmHg)**	68.2 ± 9.4	71.2 ± 10.4	65.5 ± 6.7	p=0.122
**24h Pulse pressure (mmHg)**	46.3 ± 6.4	46.1 ± 5.3	46.7 ± 8.0	p=0.802
**Dipping (%)**	13.7 ± 6.3	11.8 ± 6.4	16.1 ± 5.6	p=0.088
**Total cholesterol (mg/dL)**	192.1 ± 42.9	192.2 ± 44.1	191.9 ± 43.5	p=0.987
**HDL-cholesterol (mg/dL)**	49.3 ± 10.8	47.9 ± 10.0	51.2 ± 12.1	p=0.452
**triglycerides (mg/dL)**	126.0 ± 55.2	129.7 ± 66.3	120.8 ± 37.3	p=0.692
**LDL-cholesterol (mg/dL)**	119.5 ± 36.7	120.5 ± 36.8	118.1 ± 38.4	p=0.871
**non-HDL-cholesterol (mg/dL)**	142.8 ± 40.3	144.3 ± 41.8	140.7 ± 40.1	p=0.827
**ApoB (mg/dL)**	92.8 ± 23.5	92.9 ± 21.4	92.6 ± 27.0	p=0.982
**Uric acid (mg/dL)**	5.6 ± 1.1	5.8 ± 0.8	5.2 ± 1.4	p=0.252
**Glycemia (mg/dL)**	90.2 ± 9.4	87.7 ± 8.9	93.5 ± 9.3	p=0.117
**HbA1C (%)**	5.5 ± 0.4	5.4 ± 0.3	5.7 ± 0.3	p=0.020
**HbA1C (mmol/mol)**	36.8 ± 3.9	35.4 ± 3.8	38.8 ± 3.2	p=0.024
**Insulin (µUI/mL)**	14.4 ± 6.4	14.8 ± 7.4	14.0 ± 5.0	p=0.775
**Creatinine (mg/dL)**	0.8 ± 0.2	0.8 ± 0.2	0.9 ± 0.2	p=0.367
**Urea (mg/dL)**	35.4 ± 7.4	34.1 ± 7.3	37.2 ± 7.5	p=0.299
**Sodium (mEq/L)**	140.2 ± 1.9	139.9 ± 1.9	140.5 ± 1.9	p=0.499
**Potassium (mEq/L)**	4.3 ± 0.3	4.2 ± 0.6	4.5 ± 0.3	p=0.081
**HS-CRP (mg/L)**	1.88 (0.79-4.03)	0.82 (0.52-3.31)	3.29 (1.71-5.49)	p=0.050
**24h sodium excretion (mEq/24h)**	184.4 ± 79.7	198.1 ± 89.6	169.0 ± 61.3	p=0.368
**24h potassium excretion (mEq/24h)**	60.4 ± 28.3	58.3 ± 26.0	67.7 ± 29.6	p=0.407

**HOMA index**	2.89 (2.06-4.51)	2.86 (1.69-3.75)	2.90 (2.09-4.36)	p=0.815
**Albuminuria (mg/g creatinuria)**	5.8 (3.03-13.6)	7.00 (2.35-12)	5.6 (4.10-14.70)	p=0.728
**24h cortisol excretion (mcg/24h)**	133.1 (72.2-276.2)	164.6 (97.4-345)	120 (41.4-272.1)	p=0.511

* p-value is for the difference between KD and MD at baseline

BMI = Body Mass Index, FFM = Free Fat Mass, FM = Fat Mass, TBW = Total Body Water, ICW = Intra-Cellular Water, ECW = Extra-Cellular Water, SBP = Systolic Blood Pressure, DBP = Diastolic Blood Pressure, HDL = High-Density Lipoprotein, LDL = Low-Density Lipoprotein, ApoB = Apolipoprotein B, HbA1C = Glycated Haemoglobin, HS-CRP = High Sensitivity C-Reactive Protein, HOMA = Homeostatic Model Assessment

Table S2. Characteristics of the overall study population at baseline and follow-up

Parameters	Overall (n = 26)		p-value
	T0	T3	
**Age (years)**	46 ± 11.9	-	-
**Sex (%, male)**	65.4%	-	-
**Body weight (kg)**	96.6 ± 15.0	86.8 ± 15.8	p<0.001
**BMI (kg/m^2)**	32.9 ± 3.7	29.5 ± 4.2	p<0.001
**Waist circumference (cm)**	107.4 ± 9.4	97.4 ± 11.6	p<0.001
**FFM (kg)**	61.7 ± 13.2	57.8 ± 12.6	p<0.001
**FFM (%)**	63.7 ± 7.8	66.5 ± 7.8	p<0.001
**FM (kg)**	34.9 ± 8.4	29.1 ± 8.6	p<0.001
**FM (%)**	36.3 ± 7.8	33.5 ± 7.8	p<0.001
**TBW (l)**	44.5 ± 9.5	41.7 ± 9.1	p<0.001
**TBW (%)**	45.9 ± 5.6	48.0 ± 5.6	p<0.001
**ICW (l)**	23.5 ± 5.4	21.9 ± 4.9	p<0.001
**ICW (%)**	52.8 ± 3.7	52.4 ± 3.3	p=0.444
**ECW (l)**	20.9 ± 4.7	19.8 ± 4.6	p=0.001
**ECW (%)**	47.2 ± 3.7	47.6 ± 3.3	p=0.444
**SBP (mmHg)**	134.9 ± 9.3	123.2 ± 11.1	p<0.001
**DBP (mmHg)**	87.9 ± 6.8	80.0 ± 8.2	p<0.001
**24h mean SBP (mmHg)**	125 ± 11.3	116.1 ± 8.5	p=0.003
**24h mean DBP (mmHg)**	79.0 ± 8.4	73.7 ± 6.4	p<0.001
**Day-time mean SBP (mmHg)**	130.8 ± 10.9	121.5 ± 9.7	p=0.003
**Day-time mean DBP (mmHg)**	83.3 ± 9.0	78.1 ± 6.6	p=0.001
**Night-time mean SBP (mmHg)**	112.5 ± 13.3	105.4 ± 8.7	p=0.011
**Night-time mean DBP (mmHg)**	68.2 ± 9.4	64.5 ± 6.8	p=0.017
**24h Pulse pressure (mmHg)**	46.3 ± 6.4	39.2 ± 12.6	p=0.022
**Dipping (%)**	13.3 ± 6.0	12.8 ± 6.4	p=0.711
**Total cholesterol (mg/dl)**	192.1 ± 42.9	172.3 ± 38.7	p=0.012
**HDL-cholesterol (mg/dl)**	49.3 ± 10.8	47.2 ± 11.1	p=0.175
**Triglycerides (mg/dl)**	126.0 ± 55.2	93.7 ± 41.6	p=0.016
**LDL-cholesterol (mg/dl)**	119.5 ± 36.7	106.3 ± 37.0	p=0.046
**Non-HDL-cholesterol (mg/dl)**	142.8 ± 40.3	124.2 ± 39.0	p=0.014
**ApoB (mg/dl)**	92.8 ± 23.5	86.7 ± 22.4	p=0.172
**Uric acid (mg/dl)**	5.6 ± 1.1	5.8 ± 1.4	p=0.173
**Glycemia (mg/dl)**	90.2 ± 9.4	84.2 ± 9.2	p=0.002
**HbA1c (mmol/mol)**	36.8 ± 3.9	35.2 ± 7.0	p=0.169

**Insulin (µUI/ml)**	14.4 ± 6.4	9.8 ± 6.4	p=0.002
**Creatinine (mg/dl)**	0.8 ± 0.2	0.8 ± 0.2	p=0.723
**Urea (mg/dl)**	35.4 ± 7.4	34.7 ± 9.8	p=0.594
**Sodium (mEq/l)**	140.2 ± 1.9	141.2 ± 2.7	p=0.042
**Potassium (mEq/l)**	4.3 ± 0.3	4.4 ± 0.4	p=0.389
**HS-CRP (mg/dl)**	1.88 (0.79-4.03)	1.29 (0.43-3.30)	p=0.408
**24h Na excretion (mEq/24h)**	184.4 ± 79.7	145.4 ± 77.3	p=0.102
**24h K excretion (mEq/24h)**	60.4 ± 28.3	66.8 ± 31.7	p=0.498
**HOMA index**	2.89 (2.06-4.51)	1.73 (1.15-2.76)	p=0.001
**Albuminuria/Creatininuria (mg/g)**	5.8 (3.03-13.6)	-	-
**24h Cortisol excretion (mcg/24h)**	133.1 (72.2-276.2)	-	-

BMI = Body Mass Index, FFM = Free Fat Mass, FM = Fat Mass, TBW = Total Body Water, ICW = Intra-Cellular Water, ECW = Extra-Cellular Water, SBP = Systolic Blood Pressure, DBP = Diastolic Blood Pressure, HDL = High-Density Lipoprotein, LDL = Low-Density Lipoprotein, ApoB = Apolipoprotein B, HbA1C = Glycated Haemoglobin, HS-CRP = High Sensitivity C-Reactive Protein, HOMA = Homeostatic Model Assessment

Table S3. Correlations between the 24-hour, daytime and night-time systolic blood pressure changes and the variation in anthropometric, body composition and metabolic parameters

Parameters	Pearson's Rho	p-value
24-hour SBP		
ΔWeight (Kg)	0.288	0.172
ΔBMI (Kg/m^2)	0.292	0.166
ΔWC (cm)	0.155	0.469
ΔFFM(Kg)	0.153	0.476
ΔFFM (%)	-0.343	0.101
ΔFM (Kg)	0.320	0.127
ΔFM (%)	0.345	0.099
ΔTBW (L)	0.150	0.485
ΔTBW (%)	-0.332	0.113
ΔICW (L)	0.123	0.567
ΔICW (%)	0.120	0.578
ΔECW (L)	0.052	0.81
ΔECW (%)	-0.120	0.578
ΔHOMA	-0.183	0.392
ΔInsulin (µUI/L)	-0.089	0.68
ΔGlucose (mg/dL)	-0.303	0.15
Δ24h Na excretion (mEq/24h)	0.074	0.715
Daytime SBP		
ΔWeight (Kg)	0.167	0.435
ΔBMI (Kg/m^2)	0.182	0.394
ΔWC (cm)	0.026	0.902
ΔFFM(Kg)	-0.026	0.903
ΔFFM (%)	-0.367	0.078
ΔFM (Kg)	0.303	0.150
ΔFM (%)	0.367	0.078
ΔTBW (L)	-0.020	0.925
ΔTBW (%)	-0.360	0.084

**ΔICW (L)**	-0-019	0.928
**ΔICW (%)**	0.079	0.712
**ΔECW (L)**	-0.021	0.923
**ΔECW (%)**	-0.079	0.712
**ΔHOMA**	-0.137	0.524
**ΔInsulin (µUI/L)**	-0.122	0.569
**ΔGlucose (mg/dL)**	-0.214	0.315
**Δ24h Na excretion (mEq/24h)**	0.081	0.728
**Night-time SBP**		
**ΔWeight (Kg)**	0.176	0.412
**ΔBMI (Kg/m^2)**	0.167	0.436
**ΔWC (cm)**	-0.007	0.972
**ΔFFM(Kg)**	0.046	0.832
**ΔFFM (%)**	-0.248	0.243
**ΔFM (Kg)**	0.263	0.215
**ΔFM (%)**	0.248	0.243
**ΔTBW (L)**	0.050	0.818
**ΔTBW (%)**	-0.242	0.255
**ΔICW (L)**	-0.093	0.666
**ΔICW (%)**	-0.097	0.652
**ΔECW (L)**	0.137	0.524
**ΔECW (%)**	0.097	0.652
**ΔHOMA**	-0.266	0.208
**ΔInsulin (µUI/L)**	-0.269	0.204
**ΔGlucose (mg/dL)**	-0.300	0.154
**Δ24h Na excretion (mEq/24h)**	0.041	0.860

SBP = Systolic Blood Pressure, BMI = Body Mass Index, FFM = Free Fat Mass, FM = Fat Mass, TBW = Total Body Water, ICW = Intra-Cellular Water, ECW = Extra-Cellular Water, HOMA = Homeostatic Model Assessment
